# Examining the Choroid in Ocular Inflammation: A Focus on Enhanced Depth Imaging

**DOI:** 10.1155/2014/459136

**Published:** 2014-06-16

**Authors:** Abeir Baltmr, Sue Lightman, Oren Tomkins-Netzer

**Affiliations:** ^1^Moorfields Eye Hospital, City Road, London, EC1V2PD, UK; ^2^UCL Institute of Ophthalmology, 11-43 Bath Street, London EC1V 9EL, UK

## Abstract

The choroid is the vascular layer that supplies the outer retina and is involved in the pathogenesis of several ocular conditions including choroidal tumors, age related macular degeneration, central serous chorioretinopathy, diabetic retinopathy, and uveitis. Nevertheless, difficulties in the visualization of the choroid have limited our understanding of its exact role in ocular pathology. Enhanced depth imaging optical coherent topography (EDI-OCT) is a novel, noninvasive technique that is used to evaluate choroidal thickness and morphology in these diseases. The technique provides detailed objective *in vivo* visualization of the choroid and can be used to characterize posterior segment inflammatory disorders, monitor disease activity, and evaluate efficacy of treatment. In this review we summarize the current application of this technique in ocular inflammatory disorders and highlight its utility as an additional tool in monitoring choroidal involvement in ocular inflammation.

## 1. Introduction

**Figure 1:**
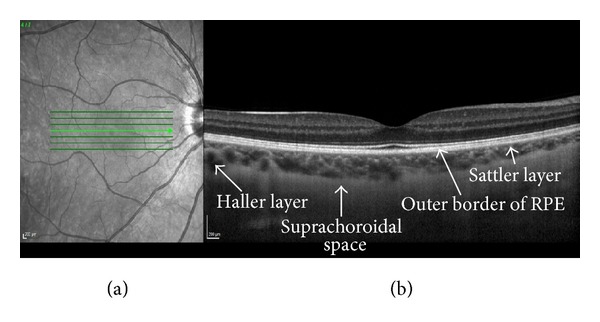
Enhanced depth optical coherence tomographic B-scan using a Heidelberg Spectralis OCT (Heidelberg Engineering, Germany). (a) Near-infrared fundus image, (b) corresponding EDI-OCT demonstrating normal retinal and choroidal anatomy at the macula. Note that both retinal and choroidal layers can be clearly identified on the same scan.

The choroid is the posterior portion of the uveal tract and outlines the retina and retinal pigment epithelium (RPE) [1]. It comprises three vasculature layers: Haller layer that includes large vessels, Sattler layer with medium vessels occupying choroidal stroma (Figure 1), and the innermost layer of choriocapillaris that is in contact with Bruch's membrane [1]. It provides up to 85% of the ocular blood flow and is solely responsible for the blood supply to the outer two thirds of the retina [1, 2].

The choroid has been implicated in the pathogenesis of many posterior segment inflammatory disorders, including Vogt-Koyanagi-Harada syndrome (VKH) [3], Behçet's disease [4, 5], sarcoidosis [6, 7], birdshot chorioretinopathy [8, 9], sympathetic ophthalmia [10, 11], panuveitis [12], toxoplasmosis [13], and posterior scleritis [14]. Due to the location of the choroid under the RPE most clinically available imaging modalities including fundus fluorescein angiogram (FFA), B-Scan ultrasonography, and optical coherence tomography (OCT) provide only partial information regarding its structure and function. This is mainly due to signal loss and light scattering at the RPE layer that is highly reflective and blocks most signals from the choroid [15].

Indocyanine green angiography (ICGA) is a technique that uses tricarbocyanine dye to visualize the choroid and delineate the choroidal circulation [16, 17]. Despite its role in evaluating inflammatory lesions in several conditions [8, 17, 18], its use is limited due to the fact that it is an invasive procedure [17]. Unlike OCT, ICGA lacks depth information and does not provide cross-sectional images of the choroid. These problems limit its role in patients' follow-up [19].

## 2. Enhanced Depth Imaging Optical Coherence Tomography (EDI-OCT) 

OCT uses the principle of low coherence interferometry to obtain in-depth information from various retinal structures to create cross-sectional images. It is an extremely useful tool for visualizing and defining different retinal layers and it helps identify retinal pathology. However, OCT is limited to imaging the retina and optic nerve head and generally cannot penetrate the RPE. Recent developments have improved its capability in imaging deeper structures, including the choroid. This technique, term enhanced depth imaging (EDI) (Figure 1), provides detailed information of the choroid by displacing the zero delay point, which is the point of maximal OCT signal sensitivity. Placing the zero delay point closer to the choroid rather than the inner retinal layers results in an enhanced visualization of the choroid and enables quantitative measurement of its thickness with high reliability and reproducibility [15, 20, 21].

## 3. Choroidal Thickness

Choroidal thickness is measured by calculating the distance from the hyperreflective line representing the outer border of the RPE (Figure 1) to the inner edge of the suprachoroidal space, which is represented by a hyporeflective line on the EDI-OCT (Figure 1). Although choroidal thickness is routinely measured manually using the digital caliper of the machine [15, 22] or by a validated custom image grading software [23, 24], automated software is available [25]. Using EDI, the mean subfoveal choroidal thickness in normal individuals has been estimated to be between 287 *μ*m and 335 *μ*m [15, 26, 27]. The variation in choroidal thickness is probably due to several variables such as gender, where choroidal thickness in men was found to be 62 *μ*m greater than in women [28]; age, with progressive subfoveal choroidal thinning at a rate of 15.6 *μ*m per decade [26, 29]; axial length and the refractive state of the eye also affecting choroidal thickness with each diopter of myopia resulting in a 8.7 *μ*m reduction in choroidal thickness [30]. Topographic variation in choroidal thickness also occurs with the maximal thickness at the subfoveal area and the thinnest nasally and inferiorly [23, 26].

The EDI-OCT technique has been used to evaluate the choroid in cases of choroidal tumors [31], diabetic macular oedema [32], glaucoma [33], age related macular degeneration [34], central serous chorioretinopathy [35], and age related choroidal atrophy [29]. In this paper we look at the application of EDI-OCT in ocular inflammatory disorders and highlight its potential in monitoring choroidal changes, which may provide an additional tool for better management in these disorders.

## 4. EDI-OCT Scan in Ocular Inflammatory Disorders

### 4.1. EDI-OCT in Noninfectious Uveitis 

#### 4.1.1. Vogt-Koyanagi-Harada Disease

Vogt-Koyanagi-Harada disease (VKH) is a multisystemic, granulomatous inflammatory disorder with presumed T-cell mediated autoimmune dysregulation towards melanocytes. The disease has four clinical phases, prodromal, acute, chronic (convalescent), and chronic recurrent, and is characterized by ocular, dermatological, and neurological involvement [3]. In the eye it is characterized by bilateral granulomatous panuveitis that initially presents as diffuse choroiditis with multifocal serous detachments that may coalesce into an exudative retinal detachment. Later during the course of the disease, signs of chorioretinal depigmentation, sunset glow fundus, or perilimbal vitiligo (Sugiura sign) are seen. Patients may also develop recurrent or chronic anterior uveitis during the chronic stage of the disease [36].

**Figure 2:**
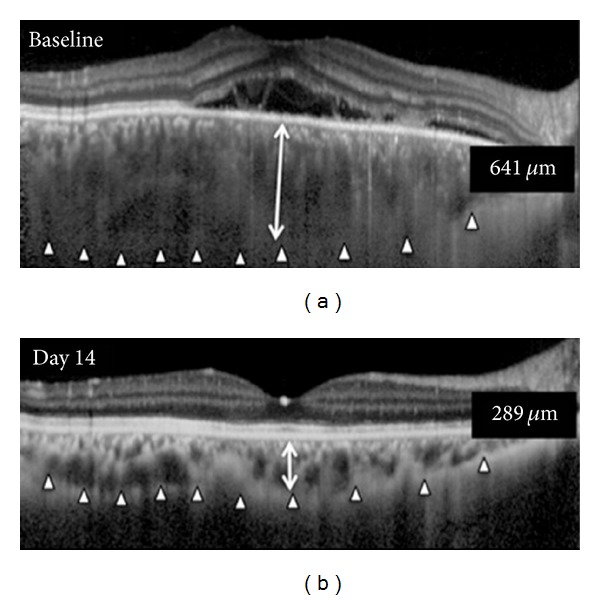
Enhanced depth optical coherence tomographic B-scan of the right eye of a 35-year-old patient with VKH taken using Heidelberg Spectralis OCT (Heidelberg Engineering, Germany). Arrowheads delineate the outer border of the choroid. At baseline subfoveal choroidal thickness of 641 *μ*m with serous retinal detachment, this was reduced with steroid therapy by day 14 to 289 *μ*m with resolution of serous retinal detachment. (Reprinted with permission from “Subfoveal choroidal thickness after treatment of Vogt-Koyanagi-Harada disease” [37].)

In a study of EDI-OCT scans during the acute stage of VKH a marked increase in the average subfoveal choroidal thickness was found in sixteen eyes of eight patients (805 ± 173 *μ*m). Following systemic steroid therapy and resolution of the inflammation, this declined by day fourteen to 341 ± 70 *μ*m [37]. An EDI-OCT of a representative case from this cohort is illustrated in Figure 2 demonstrating an enlargement of the subfoveal choroidal thickness during acute VKH with subsequent resolution with treatment.

In a second study of five eyes of patients with new onset VKH, following reduction of choroidal thickness with treatment, EDI-OCT was useful in detecting rebound choroidal thickness described as an increase of more than 100 *μ*m in the absence of other clinical signs of inflammation [38]. Morphological changes in the choroid of VKH patients were also described by another group who looked at twelve eyes of six patients with acute and chronic VKH. The authors reported a significant increase in choroidal thickness of 424 ± 50.1 *μ*m during the acute stage of the disease and a loss of the hyperreflective dots in the inner choroid during both acute and chronic phases, which may reflect changes in choroidal vasculature that occur with inflammation [39]. The role of EDI-OCT in detecting subclinical recurrence after resolution of the acute inflammation was demonstrated in a 71-year-old patient who presented six months after his initial diagnosis of VKH with headache, tinnitus, bilateral sensorineural hearing loss, and rebound choroidal thickening in the absence of other signs of ocular inflammation. The patient was taking 5 mg oral prednisolone on alternate days at presentation and increasing the dose to a 100 mg per day led to a speedy resolution of the hearing loss and a reduction in the choroidal thickness [40].

Ocular depigmentation that appears as sunset glow fundus is a predominant feature in the chronic stage of VKH [36]. In a study that looked at 19 patients with chronic VKH who were in remission with no immunosuppressive treatment for over 3 years, the subfoveal choroidal thickness was found to be significantly and inversely correlated with the amount of fundus pigmentation, the area of peripapillary atrophy, and the duration of the disease. The mean subfoveal choroidal thickness was 144 ± 72 *μ*m in patients with severe depigmentation of the fundus, 249 ± 35 *μ*m in patients with no or mild depigmentation of the fundus, and 227 ± 58 *μ*m in the controls [41]. A similar finding of progressive choroidal thinning in chronic VKH was also observed by another group, in a cohort of 16 patients with VKH [42]. EDI-OCT demonstrates that in VKH the choroid seems to thicken at times of active acute inflammation and reduces in thickness during the chronic phase. It provides a noninvasive tool to monitor disease activity and help treatment decisions.

#### 4.1.2. Behçet's Disease

Behçet's disease (BD) is an idiopathic multisystemic disease that affects many organs and is characterized by oral, genital mucocutaneous ulceration, skin lesions, and uveitis. The disease is most prevalent in the Mediterranean region as well as Japan and is associated with the HLA-B5 allele. Ocular BD is frequently bilateral and mainly presents with acute bilateral nongranulomatous panuveitis, occlusive diffuse vasculitis, retinitis, and vitritis [43].

Two studies have evaluated choroidal thickness in BD using EDI-OCT scans. The first one looked at 30 eyes from 30 Korean patients and compared the subfoveal choroidal thickness in these eyes during the active and quiescent phases of posterior uveitis. Subfoveal choroidal thickness during the acute stage (398.77 ± 155.59 *μ*m) was significantly greater than the quiescent phase of the disease (356.72 ± 141.09 *μ*m) and significantly correlated with the amount of leakage on the FFA [22]. This was attributed to choroidal infiltration by inflammatory cells and matched earlier immunohistopathological studies [4]. Interestingly, during the quiescent phase of the disease, defined as no clinical signs of inflammation in the eye for at least three months with resolution of vascular leakage on the angiogram, subfoveal thickness was still significantly greater than in healthy controls (259.96 ± 65.16 *μ*m). This possibly reflects persistent subclinical inflammation, and further investigations such as FFA and ICGA may be needed. Also of interest was a lack of significant difference in choroidal thickness between the two eyes in patients with unilateral BD, raising the possibility of choroidal infiltration of the fellow, clinically uninvolved eye during disease exacerbations. No significant correlation between choroidal thickness and the duration of the disease was observed in this study [22].

A second study that was conducted in Turkey compared the subfoveal choroidal thickness between BD patients without ocular involvement and those with BD uveitis during the active and quiescent phases of inflammation. The study looked at the subfoveal choroidal thickness in 35 eyes from 35 patients with BD posterior uveitis (289 ± 74 *μ*m), 35 eyes from 35 BD with no ocular involvement (337 ± 88 *μ*m), and 30 eyes of healthy individuals that were used as controls (329 ± 64 *μ*m). This relative thinning in eyes of BD posterior uveitis patients may be attributed to progressive fibrosis and thinning of the choroid that usually happens during the first 2-3 years of the BD, possibly due to choroidal ischemic changes from the recurrent inflammation [44]. These findings might be due to different cohorts at various stages of the disease and perhaps suggest the need for future studies to further explore choroidal activity during BD.

#### 4.1.3. Sarcoidosis

Sarcoidosis is a T-cell mediated, multisystemic, granulomatous inflammatory disorder of unknown etiology. The ocular presentation of sarcoidosis is variable and manifests as anterior, intermediate, or panuveitis. Multifocal choroiditis, choroidal and optic disc granulomas, and segmental and rarely occlusive phlebitis may also be seen [45, 46].

There is only one case report on the morphological characterization of a choroidal sarcoid granuloma using EDI-OCT in a 63-year-old patient with biopsy proven systemic sarcoidosis. The choroidal granulomas were seen as homogenous, hyporeflective demarcated lesions that reduced in size with commencement of immunosuppressant treatment [47]. Healthy choroidal tissues between these lesions and subretinal fluid adjacent to the peripapillary choroidal lesions were demonstrated [47]. This indicates the prospective of EDI-OCT in evaluating morphological changes of the choroid in ocular sarcoidosis and demonstrating response to treatment.

#### 4.1.4. Birdshot Chorioretinopathy

Birdshot chorioretinopathy (BSCR) is an idiopathic bilateral chorioretinopathy, characterized by deep oval, creamy white indistinct choroidal lesions. These lesions radiate from the disc towards the equator and are frequently associated with mild vitritis. The majority of affected patients have a positive HLA-A29 allele and FFA usually shows venous hyperfluorescence with extensive late intraretinal and disc leakage [9].

In a study of twenty four eyes of twelve patients with clinically active or quiescent BSCR, both macular and extramacular EDI-OCT scans were taken and compared to healthy controls [23]. BSCR patients were found to have a generalized retinal thinning with loss of the photoreceptor inner-outer segment junction, significant subfoveal choroidal thinning (276 ± 101 *μ*m) in comparison with controls (337 ± 74 *μ*m), absence or thinning of the Sattler vessel layer, and extramacular choroidal thinning [23], which could be attributed to late stage of BSCR. The clinically observed chorioretinal lesions corresponded to hyporeflective spots due to choroidal depigmentation and were surrounded by choroidal vessels on the EDI-OCT scan. Some patients also displayed discrete hyperreflective spots surrounding the BSCR lesions, thought to represent either pigmentary or inflammatory cellular infiltrate [23]. On ICG, BSCR lesions were identified as dark hypofluorescent spots with a tendency of active lesions to become isofluorescent during the late phase of the ICG [8]. This suggests EDI-OCT may be used as a noninvasive tool to monitor choroidal involvement in BSCR.

#### 4.1.5. Sympathetic Ophthalmia (SO)

Sympathetic ophthalmia is a granulomatous panuveitis typically occurring after penetrating trauma or surgery to one eye (the exciting eye), that eventually threatens the vision in the contralateral eye (the sympathizing eye) [10].

In a 39-year-old male, who had a penetrating injury to his left eye associated with uveal tissue prolapse, an EDI-OCT scan was useful in delineating the choroidal inflammation and the response to therapy. This patient presented one month after his initial trauma with blurred vision in the right eye [48]. Wide-field FFA showed multiple pinpoint areas of leakage at the right posterior pole, and an EDI-OCT scan revealed subfoveal fluid and a choroidal thickness of more than 500 *μ*m in that eye. Following treatment with prednisolone at 60 mg per day a reduction of choroidal thickness in the right eye to 237 *μ*m was noted. The diagnosis of SO was confirmed by histopathological examination of the left eye after elective enucleation. In this case, reduction in choroidal thickness on EDI-OCT was valuable in monitoring the response to treatment, which was in keeping with an improvement in vision [48].

#### 4.1.6. Idiopathic Panuveitis

Panuveitis is an intraocular inflammation that affects the anterior chamber, vitreous, retina, and/or choroid [49]. Panuveitis can be associated with several diseases; however, a relatively large number of cases remain idiopathic [12]. In a study that compared 21 eyes of 21 patients with inactive idiopathic panuveitis to healthy controls, the severity of the disease was correlated with the degree of choroidal thinning where patients had average choroidal thickness of 233.7 ± 73.3 *μ*m, which was thinner than controls. This was attributed to thinning of Haller's vessel layer and hyporeflectivity that is possibly due to loss of luminal spaces in choroidal vasculature, which might implicate Haller's layer in the pathophysiology of idiopathic panuveitis [24]. EDI-OCT may provide an accurate way to further understand the role of choroid in idiopathic panuveitis.

### 4.2. EDI-OCT in Infectious Uveitis

#### 4.2.1. Toxoplasma Retinochoroiditis

*Toxoplasma gondii* is the commonest cause of posterior uveitis in immunocompetent patients [50]. Reactivation of toxoplasmosis is characterized by focal retinitis adjacent to an old scar and is usually associated with vitritis that can be severe [51].

Choroidal changes in patients with toxoplasma retinochoroiditis were evaluated in 19 eyes of 15 patients with primary or reactivated toxoplasmosis. During the active stage there was a marked increase in choroidal thickness under the active lesion, demonstrated by increased hyporeflectivity on EDI-OCT. This was thought to be secondary to thickening of the retinal layers. The mean choroidal thickness declined from 390 ± 245 *μ*m during the active stage of the disease to 189 ± 86 *μ*m at last follow-up. No change in subfoveal choroidal thickness was observed during any phases of disease [52]. During the inactive phase of toxoplasmosis, four types of retinochoroidal scars were identified, atrophic scars that were associated with choroidal thinning, elevated retinochoroidal scaring associated with normal choroidal thickness, combined scars (atrophic + elevated) with mixed features of both, and deep scars that were associated with significant thinning of the choroid with loss of normal choroidal architecture [52]. These findings suggest the potential of EDI-OCT in morphological characterization of the choroidal and retinal changes in ocular toxoplasmosis.

#### 4.2.2. Fungal Choroidal Granuloma

Endogenous fungal endophthalmitis is a devastating intraocular infection that haematogenously spreads to the eye from a distant source of infection [53]. Painful reduction of vision and photophobia are the usual presenting symptoms due to either anterior or posterior segment inflammation. It is a potentially blinding condition and the rate of visual loss was found to be higher with* Candida* species [54]. There are several predisposing factors for developing fungus endophthalmitis including systemic diseases such as diabetes mellitus malignancies, organ failures, and transplantation. Recent hospitalization with long-term indwelling lines, immunosuppressive therapy, intravenous drug abuse, and intraocular surgeries are also related to an increased risk [53]. Nevertheless, the disease has also been reported in immunocompetent patients [55, 56]. In a 58-year-old immunocompetent patient, who presented with sudden painful uniocular blurred vision and panuveitis with a patch of chorioretinitis, a well demarcated choroidal mass was detected on EDI-OCT scans. A positive aqueous tap for panfungal genome confirmed the diagnosis of fungal choroidal granuloma and the choroidal lesion responded and reduced in size following successful antifungal treatment with fluconazole [56]. This case suggests the benefit of EDI-OCT scans in assessing such patients and confirming response to treatment [56].

### 4.3. EDI-OCT in Posterior Scleritis

Scleritis is a serious, painful, and potentially blinding inflammation that affects the sclera. The disease can involve the anterior or the posterior sclera and may have local or systemic associations. Posterior scleritis may present with serous retinal detachments, optic disc swelling, or choroidal effusions [57].

The choroid, being in a close apposition to the sclera, is found to be thickened during acute attacks and thinned after repeated episodes of posterior scleritis. In two cases with new onset acute noninfectious posterior scleritis a marked thickening of the choroid was noted [58]. In a 58-year-old patient who presented with unilateral pain and serous retinal detachment, the subfoveal choroidal thickness in the affected eye was found to be 548 *μ*m. Following systemic steroid treatment, choroidal thickness reduced to 308 *μ*m at two weeks and to 226 *μ*m at six months. In a second case, a 65-year-old patient presented with bilateral ocular redness and pain. The subfoveal choroidal thickness was 447 *μ*m in the right eye and 446 *μ*m in the left eye, and after treatment with systemic steroids it reduced to 393 *μ*m in the right eye and 375 *μ*m in the left eye at two weeks. By two months the sclera reduced in thickness to 372 *μ*m in the right eye and 374 *μ*m in the left eye [58]. Interestingly, in two other cases of young patients with unilateral acute recurrent posterior scleritis the choroid was significantly thinner than the unaffected eye [59]. Recurrent inflammatory changes in the sclera are thought to induce atrophic changes in the choroid resulting in progressive choroidal thinning. In a 33-year-old-patient with recurrent unilateral posterior scleritis, who was symptom-free for over two years on a maintenance dose of immunosuppression, following a relapse he presented with ocular pain and serous retinal detachment with a subfoveal choroidal thickness of 220 *μ*m. The subfoveal choroidal thickness in the contralateral uninvolved eye was 375 *μ*m. Increasing the immunosuppression was associated with resolution of symptoms and reduction of choroidal thickness in the involved eye to 143 *μ*m while the choroidal thickness in the contralateral eye measured 390 *μ*m at 35-month follow-up. A similar finding was observed in a second young patient who relapsed 53 months after his initial presentation with a unilateral serous retinal detachment and presented with a subfoveal choroidal thickness of 235 *μ*m in the affected eye and 374 *μ*m in the uninvolved contralateral eye. This reduced with restarting prednisolone treatment to 198 *μ*m while the other eye maintained the same subfoveal choroidal thickness of 374 *μ*m at 59-month follow-up. These findings suggest the possibility of monitoring severity of inflammation and the response to treatment during acute attacks of posterior scleritis as well as during relapses.

## 5. Conclusion

EDI-OCT is a noninvasive reproducible technique that allows enhanced visualization and* in vivo *measurement of choroidal thickness that could be superior to B-scan ultrasound, which has low resolution and can be less reliable when used by inexperienced examiners. Though it may be difficult to delineate the inner edge of the suprachoroidal space, especially during acute inflammation, choroidal thickness remains a promising parameter that can be used to characterize different disease entities and monitor resolution of posterior pole inflammatory disorders and efficacy of treatment. The exact behavior of the choroid in these conditions remains unclear and further prospective studies are required to help us clarify its role in the pathogenesis of these disorders.
